# Abnormal function of the vasopressin-cyclic-AMP-aquaporin2 axis during urine concentrating and diluting in patients with reduced renal function. A case control study

**DOI:** 10.1186/1471-2369-11-26

**Published:** 2010-10-05

**Authors:** Erling B Pedersen, Ingrid M Thomsen, Thomas G Lauridsen

**Affiliations:** 1 Department of Medical Research, Holstebro Hospital, Laegaardvej 12, 7500 Holstebro, Denmark; 2 Department of Medicine, Holstebro Hospital, Holstebro, Denmark; 3 University of Aarhus, Aarhus, Denmark

## Abstract

**Background:**

The kidneys ability to concentrate and dilute urine is deteriorated during progressive renal insufficiency. We wanted to test the hypothesis that these phenomena could be attributed to an abnormal function of the principal cells in the distal part of the nephron.

**Methods:**

Healthy control subjects and patients with chronic kidney diseases were studied. Group 1 comprised healthy subjects, n = 10. Groups 2-4 comprised patients with chronic kidney disease (Group 2, n = 14, e-GFR ? 90 m1/min; Group 3, n = 11, 60 m1/min ? e-GFR < 90 ml/min; and Group 4, n = 16, 15 ml/min ? e-GFR < 60 ml/min). The subjects collected urine during 24 hours. A urine concentrating test was done by thirsting during the following 12 hours. Thereafter, a urine diluting test was performed with a water load of 20 ml/kg body weight. The effect variables were urinary excretions of aquaporin2 (u-AQP2), cyclic-AMP (u-c-AMP), urine volume (UV), free water clearance (C_H2O_), urine osmolarity (u-Osm), and plasma arginine vasopressin (p-AVP).

**Results:**

After fluid deprivation, u-Osm increased. In all groups, UV and C_H2O _decreased and u-AQP2 and u-c-AMP increased in Groups 1 and 2, but were unchanged in Group 3 and 4. P-AVP was significantly higher in Group 4 than in the other groups. During urine diluting, UV and C_H2O _reached significantly higher levels in Groups 1-3 than Group 4. Both before and after water loading, u-AQP2 and p-AVP were significantly higher and u-c-AMP was significantly lower in Group 4 than the other groups. Estimated-GFR was correlated negatively to p-AVP and positively to u-c-AMP.

**Conclusions:**

Patients with moderately severe chronic kidney disease have a reduced renal concentrating and diluting capacity compared to both patients with milder chronic kidney disease and healthy control subjects. These phenomena can be attributed, at least partly, to an abnormally decreased response in the AVP-c-AMP-AQP2 axis.

ClinicalTrials.Gov Identifier: NCT00313430

## Background

The capacity of the kidneys to concentrate and dilute urine is an important mechanism to maintain constant plasma osmolarity of the body fluid compartments. Defects in both urine concentrating and diluting ability have been measured in chronic kidney diseases, and in diseases outside the kidneys associated with either fluid retention as heart failure, liver cirrhosis and syndrome of inappropriate antidiuretic hormone secretion or dehydration as diabetes insipidus [1-3]. In addition, urine diluting capacity is reduced in hypothyreoidism and adrenal insufficiency with up regulation of AQP2 [4-6], and urine concentration capacity is reduced in thyrotoxicosis and glucocorticoid excess with down regulation of AQP2 [7,8].

A normal concentrating and diluting capacity demands delivery of fluid to the distal part of the nephron, hypertonicity of renal medullar interstitial tissue and intact water absorption in the collecting ducts of the nephron. In addition, GFR and filtered load are important factors in the process of urinary concentrating ability of the kidney, as they control the load delivered to the thick ascending limb, which generates and maintains a hypertonic medullary interstitium. One or more of these prerequisites might be abnormal during the development and progression of chronic kidney disease. The consequence is an abnormal water transport in the distal part of the nephron.

In the kidney, aquaporin-2 trafficking mediates water transport across the apical cell membrane in principal cells of the collecting ducts [2]. The short-term regulation by vasopressin implies activation of V2 receptors and subsequently trafficking of AQP-2 vesicles to the apical plasma membrane resulting in increased water permeability and absorption. The long-term regulation is due to a change in AQP-2 mRNA expression followed by AQP2 synthesis [2]. Mutation in the aquaporin2 gene causes nephrogenic diabetes insipidus. Thus, an abnormal up- or downregulation of the aquaporin2 water channels in the principal cells seems to be an important patophysiological factor in development of concentrating and diluting defects in progressive renal disesase [3,9-11]. However, it has never been studied to what extent the function of the principal cells is affected in patients with varying degrees of reduced renal function, when evaluated by simultaneous measurements of urinary excretions of aquaporin2 (u-AQP2) and cyclic-AMP (u-c-AMP), and plasma concentration of vasopressin (p-AVP) during urine concentrating and diluting.

In the present study, we wanted to test the hypothesis that u-AQP 2 and u-c-AMP were abnormal in chronic kidney disease Stages I-IV [12], and that these variables responded abnormal during urine concentrating and dilution tests.

We performed urine concentrating test of 12 hours duration, and urine diluting test of 5 hours duration in healthy control subjects and patients with chronic kidney diseases. The effect variables were u-AQP 2, u-c-AMP, urine volume (UV), free water clearance (C_H2O_), and p-AVP.

## Methods

### Participants

#### Healthy control subjects

Inclusion criteria: Both men and women; age 18- 65 years; body mass index < 30. Exclusion criteria: Clinical signs or history of diseases in the heart, lungs, kidneys or endocrine organs; abnormal laboratory tests (blood hemoglobin, white cell count, platelet counts, plasma concentrations of sodium, potassium, creatinine, albumine, bilirubine, alanine-aminotransferase, and cholesterol; blood glucose; and albumin and glucose in urine); malignancies; arterial hypertension (i.e. casual blood pressure > 140/90 mmHg); alcohol abuse (more than 21 drinks per week for males and more than 14 for females); medical treatment; pregnancy; breast-feeding; lack of oral contraceptive treatment to women in the fertile age; intercurrent diseases; medicine abuse; donation of blood less than 1 month before the study; and unwillingness to participate. Withdrawal criteria: Development of one or more of the exclusion criteria, and problems with blood sampling or urine collection.

#### Patients

Inclusion criteria: Both men and women; age 18- 75 years; a diagnosis of hypertension or an estimated GFR (e-GFR) between 15-90 ml/min. Exclusion criteria: heart failure; heart arrhythmias; pulmonary insufficiency; liver disease with plasma alanine aminotransferase >100 U/L; previous cerebrovascular insult; diabetes mellitus; other endocrine diseases than diabetes mellitus not satisfactorily controlled; malignancies; alcohol abuse (more than 21 drinks per week for males and more than 14 for females); abuse of medicine; pregnancy; other diseases or conditions prohibiting participation in the trial; unwillingness to participate. Withdrawal criteria: Development of one or more of the exclusion criteria, and problems with blood sampling or urine collection.

### Design

The investigation was as a case-control study.

### Ethics

The local Medical Ethics Committee approved the study (j. no. 2580-04). All participants received written information and gave their consent by signature. The study was carried out in compliance with the Helsinki Declaration.

### Recruitment

Healthy participants were recruited by advertisements in public and private institutions in Holstebro. Patients with primary hypertension or chronic renal failure were recruited from the Out-patients' Clinic, Department of Medicine, Section of Nephrology, Holstebro Hospital.

### Experimental procedure

An ambulatory 24 hours blood pressure was made before the participants began the study procedure.

The study started 36 hours before the participants arrived in the laboratory. *Part 1(Control period): *Urine was collected during 24 hours from 07.30 p.m.-07.30 p.m. The subjects maintained their normal daily activities, ate their usual diet, and had usual fluid intake. They followed their normal medical prescriptions. *Part 2: (Urine concentrating test): *From 07.30 p.m., the night before arriving in the laboratory, until 07. 30 a.m. the next day, a 12 hours urine collection was done, followed by one hour urine collection from 07.30 to 08.30, while the participants fasted and thirsted. *Part 3 (Urine dilution test): *Urine was collected in the following 6 periods: 08.30-09. 30 a. m.; 09.30-10. 30 a. m.; 10.30-11.30 a. m.; 11.30 a.m. -00.30 p. m.; 00.30-01.30 p.m.; 01.30-02.30 p.m. An oral water load (tap water, 20 ml per body kg weight) was given from 9.30- 9.45 a.m.

The participants arrived at 7.30 a.m. in the laboratory. An intravenous catheter was inserted in a vein in fossa cubiti in one arm for collecting blood samples every hour. The participants were in the supine position except during voiding, which took place in the sitting or standing position. A light meal consisting of one slice of toast with jam was served at 08.30 a.m. All morning medication was postponed to the end of the study.

Blood samples were drawn for measurements of p-AVP, p-Osm, p-creatinine every hour, starting at 08.30 a.m. In addition, blood samples were drawn for determination of b-hemoglobin, p-sodium, p-potassium, p-albumin, p-glucose, p-ALAT, and for women a pregnancy test, at 08.30 a.m.

Urine samples were analyzed for u-AQP2, u-Osm, u-creatinine, and u-c-AMP. Blood pressure and pulse rate were measured once every hour during the examination.

### Effect variables

The main effect variable was u-AQP2, and other effect variables were u-Osm, p-AVP, and u-c-AMP, UV and C_H2O_.

### Number of subjects

Using a significance level of 5% and a power of 90%, it could be calculated that the number of subjects in each group should 10-15, when the minimal relevant difference in u- AQP2 was 0.3 ng/min and SD was 0.2 ng/min.

### Measurements

*U-AQP-2 *was measured by radioimmunoassay as previously described, and antibodies were raised in rabbits to a synthetic peptide corresponding to the 15 COOH-terminal amino acids in human AQP2 to which was added an NH_2_-terminal cystein for conjugation and affinity purification [13]. Minimal detection level was 32 pg/tube. The coefficients of variation were 11.7% (inter-assay) and 5.9% (intra-assay).

*U-c-AMP *was measured using a kit obtained from R & D Systems, Minneapolis, MN, USA. Minimal detection level was 12.5 pmol/tube. The coefficients of variation were 6.9% (inter-assay) and 5.3% (intra-assay).

Blood samples for determination of *p-AVP *were centrifuged for 15 minutes at 3000 rpm at 4°C. Plasma was separated from blood cells and kept frozen at -20°C until assayed. AVP was extracted from plasma with C_18 _Sep-Pak (Water associates, Milford, MA, USA), and subsequently determined by radioimmunoassay [14]. The antibody against AVP was a gift from Professor Jacques Dürr, Miami, FL., USA. Minimal detection level was 0.5 pmol/L. The coefficients of variation were 13% (inter-assay) and 9% (intra-assay).

Plasma and urinary *osmolality *were measured by freezing-point depression (Advanced Model 3900 multisampling osmometer).

*Blood pressure *was measured with UA-743 digital blood pressure meter (A&D Company, Tokyo, Japan)

Plasma and urinary concentrations of *creatinine *was measured by routine methods at the Department of Clinical Biochemistry, Holstebro Hospital, Denmark.

The *estimated glomerular filtration rate (e-GFR) *was calculated according to the following formulas [15]:

$$\begin{array}{l} \text{Men: e-GFR (ml/min/70 kg)}=\text{88}\times \text{(145- age in years)/p-creatinin (mikromol/l)-3}\\ \text{Women: e-GFR (ml/min/70 kg)}=\text{75}\times \text{(145- age in years)/p-creatinin (mikromol/l)-3} \end{array}$$

*Free water clearance *was calculated according to the formula C_H2O _=UV*u-Osm/p-Osm, where C_H2O _is free water clearance in ml/min, UV is urine volume in ml/min, u-Osm is urine osmolarity mosmol/l, and p-Osm is plasma osmolarity in mosmol/l.

### Statistics

Statistical level of significance was P < 0.05 in all analyses. Kruskal-Wallis's test and Friedman's tests were used to analyze differences between several unpaired and paired groups, respectively. Mann-Whitney's test and Wilcoxon's signed rank test were used to analyze differences between two unpaired and paired groups, respectively. For the urine diluting test, we used A General Linear Model with Repeated Measures for comparison between and within groups. Spearman's rho test or Pearson's test were used for analysis of correlations between to variables. Multiple regression analysis was used to measure the influence of several effect variables on the amount of water excreted during urine diluting test. Values are given as medians with interquartile ranges in brackets, or as median with 25 and 75 quartiles. SPSS version 11.5 was used for the statistical analyses.

## Results

### Demographics

#### Healthy control subjects

Sixteen healthy subjects were recruited. Six subjects were excluded, five due to abnormal 24 hours ambulatory blood pressure measurement and one due to incorrect urine collection. Thus, ten subjects fulfilled the study (Group 1), five women and five men, with a median age of 41 years (range 30-54 years), a median body mass index of 23 (range 20-27) and a median e-GFR of 108 ml/min/kg (range 93-142 ml/min/kg).

#### Patients

Forty-one patients were included and fulfilled the study. The patients were classified in three groups according to e-GFR (Groups 2-4). Group 2 (n = 14) comprised 6 women and 8 men, with a median age of 49 years (range 30-58 years), a median body mass index of 26 (range 20-35) and a median e-GFR of 99 ml/min/70 kg (range 90-140 ml/min/70 kg). Group 3 (n = 11) comprised 1 woman and 10 men with a median age of 53 years (range 39-71 years), a median body mass index of 26 (range 23-34) and a median e-GFR of 79 ml/min/70 kg (range 65-89 ml/min/70 kg). Group 4 (n = 16) comprised 4 women and 12 men with a median age of 59 years (range 37-73 years), a median body mass index of 27 (range 22-32) and a median e-GFR of 25 ml/min/70 kg (range 12-56 ml/min/70 kg).

The primary renal disease was nephrosclerosis (n = 11), chronic glomerulonephritis (n = 7), adult polycystic kidney disease (n = 3), interstitial nephropathy (n = 1), and unknown nephropathy (n = 19). All patients received antihypertensive treatment in various combinations, i.e.loop diuretics (n = 11), calcium channel antagonists (n = 14), thiazides (n = 15), angiotensin converting enzyme inhibitors (n = 16), angiotensin II receptor antagonists (n = 16), β-adrenergic blockers (n = 16), α-adrenergic blockers (n = 3), and α-calcidol (n = 4).

We did not see any deterioration in renal function either during urine concentrating or diluting test.

## Urine concentration test

Table 1 shows u-Osm, UV, C_H2O_, u-c-AMP and u-AQP2 during the urine concentrating test.

**Table 1:** Urine concentrating test.

	24 hours urine collection without fluid deprivation (19.30 to 19.30)	12 hours urine collection with fluid deprivation (19.30 to 07.30)	One urine collection fluid deprivation(07.30-08.30)
**U-osm (mosmol/l)**			
Group 1	561(357)	473(344)	823(213) *
Group 2	405(286)	369(460)	670(193) *
Group 3	472(303)	411(96)	547(223) *
Group 4	359(119)	351(126)	396(153) *
P (Kruskal-Wallis)	0.169	0.188	0.000
**UV (ml/min)**			
Group 1	1.40(1.15)	1.08(1.02)	0.72(0.42) *
Group 2	1.91(1.26)	1.42(1.50)	0.97(0.94) *
Group 3	1.38(0.67)	1.69(0.41)	1.17(1.04)
Group 4	1.77(1.00)	1.84(0.67)	1.25(0.89) *
P (Kruskal-Wallis)	0.156	0.043	0.006
**C_H20 _(ml/min)**			
Group 1	-0,94(1.35)	-0,38(0.44)	-1.10(0.55)*
Group 2	-0,64(1.35)	-0,45(1.50)	-0,90(0.57) *
Group 3	-1.29(1.29)	-0,71(0.80)	-1,22(0.82)
Group 4	-0,28(0.84)	-0,13(0.73)	-0,30(0.66)
P (Kruskal-Wallis)	0.095	0.149	0.03
**U-c-AMP (pmol/l)**			
Group 1	2.53(1.53)	3.00(2.27)	4.83(4.82)*
Group 2	2.01(0.96)	2.83(2.87)	4.79(4.67) *
Group 3	2.36(0.66)	1.96(0.95)	3.65(2.45)
Group 4	1.20(1.37)	1.31(1.02)	1.20(1.17)
P (Kruskal-Wallis)	0.000	0.000	0.000
**U-AQP2 (ng/ml)**			
Group 1	0.68(0.88)	0.96(1.63)	0.72(1.26) *
Group 2	0.54(0.44)	0.51(0.98)	0.73(0.52) *
Group 3	1.13(0.47)	0.69(0.49)	0.87(0.33)
Group 4	0.79(0.63)	0.71(0.66)	0.63(0.61)
P (Kruskal-Wallis)	0.048	0.793	0.423

Urinary osmolarity (u-osm), urine volume (UV), free water clearance (C_H2O_), urinary excretions of cyclic-AMP (u-c-AMP) and aquaporin2 (u-AQP2) in a 24 hours urine collection with unrestricted fluid intake, in an immediately following 12 hours urine collection with fluid deprivation (19.30 to 07.30), and in an immediately following one hour urine collection with fluid deprivation (07.30-08.30).

Group 1 comprised healthy control subjects (n = 10). Groups 2-4 comprised patients with chronic kidney disease or hypertension (Group 2 (n = 14), e-GFR ≥ 90 ml/min; Group 3 (n = 11), 60 ml/min ≤ e-GFR < 90 ml/min; and Group 4 (n = 16), 15 ml/min < e-GFR ≤ 60 ml/min).

Median with Interquartile Range in brackets. Kruskal-Wallis's test was used to analyze differences between groups during the concentration test. Wilcoxon's signed rank test was used to analyze differences in variables between baseline (24 hours urine) and the end of the test (one hours urine), * = P < 0.05.

In all groups U-Osm increased during a 12 hours period of thirsting. The increase in u-Osm was significantly lower in Group 4 than in Group 1, and the increase was especially reduced in patients with decreased renal function (Group 1: 47%, Group 2: 65%, Group 3: 14%, and Group 4: 10%).

Urine volume decreased significantly in Groups 1 and 2 (Group 1: -49%, Group 2:- 49%), when comparison was made between the levels during 24 hours and during one hour after the 12 hours thirsting period. In addition, a tendency to decrease in UV was measured during the 12 hours thirsting period. Contrarily, in Groups 3 and 4, UV tended to increase during the 12 hours of thirsting, and at the end of the concentrating test, UV was not significantly changed in Group 3 (-15%), and was only borderline significantly reduced in Group 4 (-29%).

Free water clearances fell significantly in Group 1 (-17%) and in Group 2 (-40%), whereas the levels were approximately unchanged in Group 3 (-5%) and Group 4 (-7%), when comparison was made between the levels during 24 hours and during one hour after the 12 hours thirsting period.

The concentration of c-AMP was approximately at the same level in Groups 1-3 during 24 hours urine collection. U-c-AMP increased significantly in Group 1 (91%) and Group 2 (138%), when comparison was made between the levels during 24 hours and during one hour after the 12 hours thirsting period. There was no significant increase in u-c-AMP in groups 3 and 4, although a tendency to increase in u-c-AMP was measured in Group 3 (55%).

The concentration of AQP2 in urine increased significantly in Group 1 and 2 during the urine concentrating test. In Group 3 and 4, u-AQP2 was no significantly changed during water deprivation.

P-AVP was the same in Groups 1 to 3 (Group 1: 0.95 pmol/l (0.33), median with interquartile range, Group 2: 0.75 pmol/l (0.55), and Group 3: 1.00 pmol/l (0.83), NS). The level in Group 4 was significantly higher than in the other three groups (Group 4: 1.80 pmol/l (1.68), p < 0.000).

### Urine diluting test

Table 2 shows UV, C_H2O_, u-osm, u-c-AMP, and u-AQP2 during the urine diluting test.

**Table 2:** Urine volume (UV), free water clearance (C_H20_), urine osmolarity (u-osm), urinary excretions of cyclic AMP (u-c-AMP) and aquaporin2 (AQP2) during urine diluting test.

	Baseline	Post 0-60	Post 60-120	Post 120-180	Post 180-240	Post 240-300	P (GLM-within)
**UV (ml/min)**							
Group 1	0.8(0.4)	2.4(3.0) *	10.7(6.3)*	7.4(4.2) *	2.1(4,1) *	1.2(1.5)	0.000
Group 2	0.8(0.8)	1.9(2.8) *	7.2(5.7) *	8.2(3.1) *	3.5(2.2) *	1.3(1.8) *	
Group 3	1.1(1.0)	3.0(1.8) *	11.7(8.0)*	8.0(8.3) *	3.8(4.8) *	1.4(0.8)	
Group 4	1.3(0.9)	2.0(1.4) *	3.7(1.6) *	4.1(2.8) *	2.7(0.9) *	2.2(1.6) *	
P (GLM-between)	0.002						
P (Kruskal-Wallis)	0.016	0.869	0.000	0.001	0.246	0.016	
**C_H20 _(ml/min)**							
Group 1	-1.1(0.5)	-0.1(3.0) *	7.9(5.5) *	5.0(3.9) *	-0,43(4.0) *	-0.8(1.4)	0.000
Group 2	-0.9(0.6)	-0,4(2.5)	4.4(5.7) *	5.6(2.8) *	1.5(3.0) *	-0.3(1.9) *	
Group 3	-1.1(1.0)	-0.9(1.5)	8.7(8.4) *	5.4(8.2) *	1.3(4.3) *	-0.8(1.0) *	
Group 4	-0.3(0.6)	-0.4(0.5)	1.6(0.9) *	1.5(2.6) *	0.9(1.4) *	0.1(0.8) *	
P (GLM-between)	0.000						
P (Kruskal-Wallis)	0.003	0.264	0.000	0.001	0.309	0.017	
**U-osm (mosmol/l)**							
Group 1	709(258)	271(360)*	72(46)*	98(80)*	394(334)*	637(388)*	0.000
Group 2	678(175)	372(386)*	104(68)*	95(33)*	146(186)*	355(403)*	
Group 3	555(247)	448(285)*	86(148)*	92(122)*	219(265)*	452(216)*	
Group 4	397(145)	352(101)*	190(63)*	166(116)*	203(129)*	262(90)*	
P (GLM-between)	0.000						
P (Kruskal-Wallis)	0.000	0.484	0.000	0.001	0.100	0.027	
**U-c-AMP (μg/min)**							
Group 1	3.07(1.35)	3.83(1.77)	3.48(1.31)	3.07(1.28)	3.24(0.94)	2.97(1.48)	0.254
Group 2	3.88(2.45)	4.21(2.52)	3.91(1.56)	3.96(2.17)	3.41(3.24)	3.55(1.04)	
Group 3	3.20(1.04)	3.84(1.58)*	4.43(2.33)	3.82(2.88)	3.34(2.31)	3.00(1.31)	
Group 4	1.74(1.18)	1.99(1.34)	1.20(2.02)	1.20(1.24)	1.91(0.85)	2.50(1.33)	
P (GLM-between)	0.000						
P (Kruskal-Wallis)	0.000	0.000	0.001	0.000	0.000	0.001	
**U-AQP2 (ng/min)**							
Group 1	1.01(0.20)	1.05(0.19)	0.91(0.51)	0.78(0.27)*	0.74(0.33) *	0.74(0.33) *	0.321
Group 2	0.97(0.52)	1.04(0.63)	0.85(0.55)	0.91(0.31)	0.69(0.34) *	0.65(0.21) *	
Group 3	1.16(0.88)	1.42(0.86)	1.31(1.14)	1.16(0.67)	0.99(0.28)	0.79(0.47) *	
Group 4	1.28(0.78)	1.74(0.82)	1.40(0.52)	1.14(0.49)*	1.03(0.56) *	1.21(0.58)	
P (GLM-between)	0.002						
P (Kruskal-Wallis)	0.157	0.036	0.063	0.022	0.000	0.000	

Baseline was mean of two 60 minutes control periods before water loading. An oral water load of 20 ml/kg body weight was given during the first 15 minutes of the following 60 minute's period (Post 0-60). Urine was subsequently collected during four consecutive 60 minute's periods (Post 60-120, Post 120-180, Post 180-240, and Post 240-300. Group 1 comprised healthy control subjects (n = 10). Groups 2-4 comprised patients with chronic kidney disease or hypertension (Group 2 (n = 14), e-GFR ≥ 90 ml/min; Group 3 (n = 11), 60 ml/min ≤ e-GFR < 90 ml/min; and Group 4 (n = 16), 15 ml/min < e-GFR ≤ 60 ml/min).

Median with Interquartile Range in brackets. A General Linear Model (GLM) for Repeated Measures was used for comparison within and between groups. Kruskal-Wallis's test was used to analyze differences between groups during the diluting test. Wilcoxon's signed rank test was used in each group to analyze significant deviations from baseline (* = P < 0.05).

The amount of water given was in Group 1:1389 ml (371), in Group 2: 1581 (416), in Group 3: 1674 ml (416), and in Group 4: 1572 ml (374). The amount of water excreted during the first four hours after water loading was significantly lower in Group 4 than in the other three Groups (Group 1: 1376 ml/4 hours (465), median with interquartile range, 100% (44) of the water load, Group 2: 1210 ml/4 hours (662), 81% (30) of the water load, Group 3: 1536 ml/4 hours (524), 93% (32) of the water load, and Group 4: 797 ml/4 hours (363), 53% (17) of the water load).

Both UV and C_H2O _increased in all groups during the diluting test. UV and C_H2O _reached a significantly higher level during urine diluting in Groups 1, 2, and 3 compared with Group 4, i. e. UV (Group 1: 12 fold, Group 2: 9 fold, Group 3: 9 fold, and Group 4: 2 fold), and C_H2O _(Group 1: 9.0 ml/min, Group 2: 6.5 ml/min, Group 3: 9.8 ml/min, and Group 4: 1.9 ml/min). U-osm decreased significantly in all groups. In Group 4, u-osm was significantly higher than in the other groups during the second and third hour of the test.

U-c-AMP was unchanged during urine diluting test in all four groups. However, u-c-AMP was significantly lower in Group 4 than in the other three groups.

U-AQP2 decreased during urine diluting test in all groups. The statistical analyses showed that the level of u-AQP2 was significantly higher in Group 4 than in the other three groups during the different periods of the test. U-AQP2 was the same during the test in Groups 1, 2, and 3. The maximum decrease did not deviate significantly between the groups, u-AQP2 (Group 1: - 27%, Group 2: - 33%, Group 3: - 32%, and Group 4: - 19%).

Table 3 shows that p-AVP was significantly reduced in all four groups during urine diluting test. The maximum reduction was most pronounced in Group 1 (Group 1: - 40%, Group 2: - 11%, Group 3: - 14%, and Group 4: - 27%). The level of p-AVP was significantly higher in Group 4 than in the other three groups, in which p-AVP was approximately in the same level. P-osm decreased significantly in all groups and was normalized in the last period of the test in Groups 1 and 2.

**Table 3:** Plasma concentration of vasopressin (p-AVP) and plasma osmolaririty (p-osm) during urine diluting test.

	Baseline	Post 0-60	Post 60-120	Post 120-180	Post 180-240	Post 240-300	P (GLM-within)
**AVP (pmol/l)**							
Group 1	1.00(0.40)	0.7(0.10) *	0.60(0.25)*	0.70(0.35)*	0.90(0.65)	1.00(0.45)	0.000
Group 2	0.75(0.45)	0.60(0.08)*	0.60(0.18)*	0.60(0.10)*	0.70(0.60)	0.80(0.45)	
Group 3	0.70(0.53)	0.60(0.08)*	0.60(0.15)*	0.60(0.15)*	0.65(0.63)	0.80(0.60)	
Group 4	1.80(1.85)	1.10(0.83)*	1.10(0.88)*	1.30(0.93)*	1.50(0.85)*	1.65(1.25)*	
P (GLM-between)	0.000						
P (Kruskal-Wallis)	0.000	0.000	0.000	0.000	0.001	0.002	
**P-osm (mosmol/l)**							
Group 1	290(6)	283(3) *	284(6)*	287(5)*	286(5)*	287(4)	0.000
Group 2	288(7)	283(8)*	284(8)*	286(9)*	286(9)*	288(8)	
Group 3	291(11)	285(7)*	287(9)*	289(6)*	288(4)*	289(6)*	
Group 4	305(14)	299(15)*	298(14)*	297(14)*	297(14)*	299(13)*	
P (GLM-between)	0.000						
P (Kruskal-Wallis)	0.000	0.000	0.000	0.000	0.000	0.000	

Blood samples were drawn after a baseline period of 120 minutes, and after the end of five consecutive periods each of one hour duration (Post 0-60, Post 60-120, Post 120-180, Post 180-240, and Post 240-300. An oral water load of 20 ml/kg body weight was given during the first 15 minutes of the Post 0-60 period. Group 1 comprised healthy control subjects (n = 10). Groups 2-4 comprised patients with chronic kidney disease or hyperension (Group 2 (n = 14), e-GFR ≥ 90 ml/min; Group 3 (n = 11), 60 ml/min ≤ e-GFR < 90 ml/min; and Group 4 (n = 16), 15 ml/min < e-GFR ≤ 60 ml/min).

Median with Interquartile Range in brackets. A General Linear Model (GLM) for Repeated Measures was used for comparison within and between groups. Kruskal-Wallis's test was used to analyze differences between groups during the diluting test. Wilcoxon's signed rank test was used in each group to analyze significant deviations from baseline (* = P < 0.05).

### Blood pressure

Twenty-four hours BP was significantly different between the groups (Group 1: 123/74 (16/9.8) mm Hg, median and interquartile range, Group 2: 130/81 (12/7.0) mm Hg, Group 3:129/79 (13/8.0) mm Hg, Group 4: 140/83 (18/10) mm Hg) for both systolic and diastolic BP (0.004/0.005, Kruskal-Wallis test). The statistical analyses showed that the level in Group 4 was significantly higher than in the other groups. Pulse rate during 24 hours was the same in the groups (Group 1: 70 (13) beats/min, median and interquartile range, Group 2: 70 (12) beats/min, Group 3: 65 (15) beats/min, and Group 4: 69 (10) beats/min.

During the experimental procedure BP was measured seven times. The initial level was in Group 1: 107/62 (14/13) mm Hg, Group 2: 117/78 (24/15) mm Hg, Group 3: 123/74 (22/19) mm Hg, and Group 4: 126/77 (25/13) mm Hg. The BP and pulse levels did not deviate significantly from baseline level during the urine diluting test (Data not shown).

### Relationship between e-GFR and AVP, c-AMP and AQP2

Correlation analyses were performed for the whole study population on data from baseline before the urine diluting test. E-GFR was significantly correlated with p-AVP (ρ = - 0.618, P < 0.000), u-c-AMP (ρ = 0.536, P < 0.000), and u-AQP2 (ρ = - 0.288, P < 0.05). The influence of e-GFR on effect variables were measured using a multiple regression analysis (Table 4). The dependent variable was the amount of water excreted during the first four hours after water loading in percent of the given water load during the urine diluting test (20 ml/kg/body weight). The independent variables were p-AVP, u-c-AMP, AQP2, 24 hours systolic and diastolic blood pressure, and e-GFR. The analysis showed that the partial regression coefficient was significant for e-GFR, but not for the other effect variables in the analysis.

**Table 4:** Multiple regression analysis with excreted water as dependent variable during urine diluting test.

Independent variables	B	SE	β	t	P
P-AVP (pmol/l)	5.184	5.844	0.154	0.887	0.381
U-c-AMP (pmol/l)	-0.002	0.003	-0.93	-0.586	0.562
U-AQP2 (ng/ml)	-13.270	8.415	-0.242	-1.577	0.123
SBP (mm Hg)	0.035	0.472	0.015	0.075	0.940
DBP (mm Hg)	-0.366	0.721	-0.105	-0.507	0.615
E-GFR (ml/min)	0.487	0.159	0.613	3.066	0.004

The amount of water excreted during the first four hours after water loading in percent of the given water load during the urine diluting test (20 ml/kg/body weight) as dependent variable and plasma concentration of vasopressin (p-AVP), urinary excretions of cyclic AMP (u-c-AMP) and aquaporin2 (AQP2), 24 hours systolic and diastolic blood pressure (SBP and DBP), and estimated GFR (e-GFR) as independent variables in a multiple regression analysis at the start of the urine diluting test in the whole study population (n = 41) comprising healthy control subjects (n = 10) and patients with chronic kidney disease or hypertension (n = 31; Group 2 (n = 14), e-GFR ≥ 90 ml/min; Group 3 (n = 11), 60 ml/min ≤ e-GFR < 90 ml/min; and Group 4 (n = 16), 15 ml/min < e-GFR ≤ 60 ml/min).

Unstandardized partial regression coefficients (Β), standard error (SE), standardized partial regression coefficient (β), t- value (t), and significance (P)

## Discussion

Our study showed that patients with a renal function corresponding to chronic kidney disease stage III and IV, i. e. 15 ≤ e-GFR < 60 ml/min, have a reduced renal concentrating and diluting capacity compared to both patients with milder chronic kidney disease or hypertension, corresponding to stage I and II, i. e. GFR ≥ 60 ml/min, and healthy control subjects. Our analyses showed that these phenomena can be attributed, at least partly, to an abnormal function of the AVP-c-AMP-AQP2 axis.

In healthy subjects, AVP stimulates vasopressin receptors on the principal cells in the collecting ducts of nephron. This results in an increased production of c-AMP, which through a cascade of intracellular reactions upregulate the AQP 2 water channels, and thereby promotes water transport from the tubular lumen to the cell. Subsequently, water is further transported to the interstitial fluid through aquaporin 3 and 4 water channels [2]. In previous studies, we have demonstrated that u-AQP2 reflects the functional status of the aquaporin2 water channels during urine concentrating and diluting procedures [13,16,17]. Urinary excretion of u-c-AMP is a measure of the renal production of c-AMP. Thus, the function of the principal cells can be determined by measurements of u-AQP2 and u-c-AMP together with p-AVP.

The renal concentrating ability is a defense mechanism to prevent water depletion and hyperosmolarity in body fluids. A decrease in urinary concentrating ability can have several reasons. Firstly, the delivery of tubular fluid can be diminished due to a fall in GFR. Secondly, the ability to generate interstitial hypertonicity can be reduced due to decreased sodium and chloride absorption in the ascending limb of Henle, decreased urea accumulation, or a change in renal medullar blood flow. Thirdly, the response to vasopressin may be reduced or absent [18,19].

After the urine concentrating test, we measured a significant increase in u-Osm in all groups, and UV and C_H2O _were clearly and significantly reduced in Groups 1 and 2. However, in Groups 3 and 4 C_H2O _was unchanged, and UV was unchanged or only borderline changed in Groups 3 and 4, respectively. Thus, the increase in u-Osm and the fall in UV and C_H2O _were smaller in patients with reduced renal function, which indicated a reduced concentrating ability. Our study also showed that u-c-AMP was decreased in patients with an e-GFR < 60 ml/min, and the correlation analyses showed a significantly, positive correlation between e-GFR and u-c-AMP. Thus, the kidneys' ability to synthesize c-AMP decreased with deteriorating renal function. This phenomenon might be attributed to a reduced sensitivity to AVP, because p-AVP was increased in patients with e-GFR < 60 ml/min and correlated inversely with e-GFR in the whole group of study subjects. The concentration of AQP2 was not significantly changed during the urine concentrating test in Groups 3 and 4, but increased significantly in the other two groups. This is in good agreement with the lack of increase in u-c-AMP in Groups 3 and 4. Thus, our study showed that the reduced renal concentrating capacity during decreasing renal function can be attributed, at least partly, to a lack of responsiveness to vasopressin of the principal cells in the distal tubules. Our results are in agreement with a previous small study of patients with diabetic nephropathy, in whom a decrease in u-AQP2 was associated with impaired urinary concentrating ability [20]. In addition, our results are in good accordance with animal experiments, which have demonstrated a vasopressin resistant down regulation in expression of AQP2, when urinary concentrating ability was impaired, i. e. in nephrogenic diabetes insipidus, post-obstructive polyuria, and renal failure [1-3,9].

The renal diluting mechanism is a defense mechanism against water intoxication and hypoosmolarity.. An abnormal diluting capacity can be attributed to several factors. Firstly, a decrease of tubular fluid to the diluting segments of the nephron can be caused by a diminished GFR, an increased absorption of sodium and water in the proximal tubules or both. Secondly, an abnormally reduced absorption of sodium and chloride in the thick ascending limb of Henle or in the distal convoluted tubule can prevent establishment of sufficient interstitial hypertonicity.

Thirdly, plasma level of vasopressin may be increased due to drug treatment or primary diseases outside the kidneys as heart failure, liver cirrhosis with fluid retention, hypothyroidism and adrenal insufficiency [3]. Treatment with a vasopressin receptor antagonist reversed the consequence of non-osmotic increase in vasopressin release in these conditions and improved urinary dilution, and animal studies showed that AQP2 expression was increased [4,6].

Our study showed that urine diluting capacity was decreased in patients with moderately reduced renal function. Thus, the amount of excreted water represented more than 80% of the load during the first four hours after the load was given in Groups 1, 2 and 3, but only half of the load was excreted in Group 4. This corresponded very well with the increase in both UV and C_H2O _in all groups and a much lower increase in Group 4 than the other three groups. During urine dilution, p-AVP was reduced in all groups, but the level was significantly higher in patients with e-GFR < 60 ml/min, and p-AVP correlated significantly and inversely with e-GFR during all periods of the test. In addition, u-c-AMP was lower in Group 4 than in Groups 1, 2 and 3 and correlated significantly and positively with e-GFR during the whole test. Thus, our results showed that the high level of p-AVP in Group 4 failed to control c-AMP, presumably due to a defect function of the principal cells induced by the chronic kidney disease. This leaves the function of the AQP2 water channels without regulatory influence of the AVP-c-AMP-axis. U-AQP2 was reduced during urine dilution in all groups, but the level was higher in Group 4 that in the other three groups during the whole test. This is in accordance with a deficiency in regulatory influence on the formation of AQP2 water channels by the AVP-u-c-AMP-axis. Our results suggest that the abnormal urine dilution capacity in chronic kidney disease stage III and IV is due to a lower ability of the principal cell in the distal part of the nephron to down regulate the expression of the AQP2 water channels and in that way promote water excretion. However, it is likely that the reduced GFR and a reduced hypertonicity in the renal interstitial tissue contributed to the reduced urine diluting capacity in chronic kidney disease.

Among several variables, the multiple regression analysis demonstrated that e-GFR was the most important factor to determine the extent of disturbance in the AVP-AMP-AQP2-axis, and thus the degree of deterioration in urine concentrating and diluting ability in chronic kidney disease.

We have used established procedures to test urinary concentrating and diluting capacity [21]. Several different formulas have been suggested for estimation of GFR using p-creatinine, gender, body weight, height and ethnicity in various combinations [22]. Each of these estimates of GFR has weaknesses and strengths. In the present study, we have chosen the Hull-equation to estimate GFR [15]. We have also calculated e-GFR according to the MDRD-equations [23-25]. Using correlation analyses, we found a highly significant correlation between e-GFR estimated by the Hull-equation and both the original MDRD equation from 1999 (r = 0.978, n = 51, P < 0.000) and the new MDRD equation from 2009 (r = 0.985, n = 51, P < 0.000). Thus, it is unlikely that small differences in e-GFR from different formula would have changed the conclusions of our study, even if we had used either older or newer equations [23-25].

Urine concentrating ability is decreased in the aging mammalian kidney [26]. A functional impairment of renal concentrating ability was demonstrated in aged rats [27]. This phenomenon appeared to be related to decreased responsiveness of the kidney to vasopressin caused by a down regulation of the renal vasopressin 2 receptor and AQP2 abundance [27]. We measured a marked difference in urine concentrating and diluting ability between the three groups of patients in the present study, in spite of a rather narrow age range between the groups with medians between 49 and 59 years. Thus, it is unlikely that differences in age between the three groups of patients could explain our findings.

We studied different types of renal diseases. During progressive deterioration in renal function urinary concentrating and diluting ability may be affected differently depending on the nature of the disease, i.e. in primarily glomerular, tubular, interstitial or vascular pathogenesis. However, we demonstrated clear abnormalities in urine concentrating and diluting ability during progression to Stage III and IV despite differences in etiology of the disease.

A large proportion of the patients in the present study received antihypertensive treatment, which might influence the activity in the renin-angiotensin-aldosterone system, natriuretic peptide system, and sympathetic nervous activity. In addition to vasopressin, these homeostatic systems have a modulating influence on the transport in the aquaporin2 water channels [28-32]. It would have been ideal to perform studies after withdrawal of all antihypertensive treatment. This was not done, since we did not find it ethically justified to discontinue antihypertensive therapy. The prostaglandin system can also modulate the transport via aquaporin2 water channels, but none of the patients in the present study received non-steroid-anti-inflammatory drugs.

The present study comprised three patients with polycystic kidney disease. Animal studies indicated that vasopressin regulates cystogenesis [33], which has been basis for ongoing clinical trials regarding the effect of vasopressin antagonists on cyst growth and deterioration of renal function in patients with PKD [34]. Other ongoing trials study the effect of statins, blockade of the renin-angiotensin system, sirolimus, and somatostatins in PKD [35]. At the time being, it is far from clarified, whether these therapeutic aspects will be effective regarding cyst growth and preservation of renal function in patients with PKD or have influence on the abnormal urine concentrating and diluting ability in progressive renal disease. Neither is it known whether polycystic kidney per se will change urine concentrating and diluting ability. However, we have analyzed our data with exclusion of the three patients with PKD, and the results and conclusions were unchanged.

## Conclusions

The function of AVP-c-AMP-AQP2 axis was abnormal in patients with an e-GFR between 15 and 60 ml/min. The ability to concentrate urine was decreased in renal insufficiency due to an impaired and reduced response in c-AMP and AQP2 to AVP, which was elevated in plasma, presumably as a compensatory phenomenon. The ability to dilute urine was deteriorated in renal insufficiency, presumably due to both a lack of suppression in AVP during water loading and a defect in the regulatory function of AVP on c-AMP with a secondarily up regulation of AQP2 water channels.

## Competing interests

The authors declare that they have no competing interests.

## Authors' contributions

EBP conceived of the study, participated in design, coordination and recruitment of subjects, had responsibility for the biochemical analyses, performed most of the statistical analysis, and drafted the manuscript. IT participated in design and coordination of the study, and performed a part of the statistical analysis. TGL participated in design, coordination of the study, recruitment of subjects, and the experimental procedures. All authors read and approved the final manuscript.

## Pre-publication history

The pre-publication history for this paper can be accessed here:

http://www.biomedcentral.com/1471-2369/11/26/prepub
